# High sensitivity of Indian summer monsoon to Middle East dust absorptive properties

**DOI:** 10.1038/srep30690

**Published:** 2016-07-28

**Authors:** Qinjian Jin, Zong-Liang Yang, Jiangfeng Wei

**Affiliations:** 1 Jackson School of Geosciences, University of Texas at Austin, Austin, Texas, USA; 2 Center for Global Change Science, Massachusetts Institute of Technology, Cambridge, Massachusetts, USA

## Abstract

The absorptive properties of dust aerosols largely determine the magnitude of their radiative impacts on the climate system. Currently, climate models use globally constant values of dust imaginary refractive index (IRI), a parameter describing the dust absorption efficiency of solar radiation, although it is highly variable. Here we show with model experiments that the dust-induced Indian summer monsoon (ISM) rainfall differences (with dust minus without dust) change from −9% to 23% of long-term climatology as the dust IRI is changed from zero to the highest values used in the current literature. A comparison of the model results with surface observations, satellite retrievals, and reanalysis data sets indicates that the dust IRI values used in most current climate models are too low, tending to significantly underestimate dust radiative impacts on the ISM system. This study highlights the necessity for developing a parameterization of dust IRI for climate studies.

The Indian summer monsoon (ISM) brings approximately 70–80% of the annual rainfall in the Indian subcontinent and its anomalies (i.e., floods or droughts) often result in severe casualties and huge agricultural and economic losses in the densely populated region. The spatiotemporal variability of the ISM rainfall is attributed to various physical processes and their interactions involving the atmosphere (e.g., water vapor and circulation)^1^, ocean (e.g., sea surface temperature and El Niño–Southern Oscillation)^2^, and land surface (e.g., soil moisture and snow cover)^3^. Over the last decade, the potential impacts of aerosols on the ISM rainfall through their direct and indirect effects on these physical processes and interactions have received increasing attention^4^,^5^,^6^,^7^,^8^,^9^,^10^,^11^,^12^,^13^,^14^,^15^,^16^,^17^,^18^,^19^,^20^,^21^.

There are three main hypotheses of the dust–ISM interactions. The “solar dimming effect” hypothesizes that the anthropogenic aerosols in India could reduce the north-south land-ocean thermal contrast and increase the atmospheric stability by cooling the surface through the absorption and scattering of solar radiation, resulting in a weakened ISM^17^. This hypothesis is supported by the observed increases in anthropogenic emissions and the decrease in the ISM rainfall during the second half of the twentieth century. These changes in emissions and monsoon rainfall are also reproduced by numerical simulations^4^,^6^. In contrast, the “elevated heat pump” hypothesis states that absorptive aerosols (i.e. black carbon and mineral dust) can stack up over the Tibetan Plateau heating the mid-upper troposphere and enhancing the updraft motion, which “pumps” more moist air from south to north India, consequently strengthening ISM^13^. It should be pointed out that the “elevated heat pump” effect is subject to debate^11^,^12^,^22^,^23^. Recently, observational and modeling studies have found that the mineral dust aerosols emitted in the Middle East can strengthen the ISM system by heating the troposphere over the Iranian Plateau and the Arabian Sea^19^,^24^,^25^,^26^. This finding is further confirmed by the increasing aerosol loadings in the Middle East and an increase in ISM rainfall between 2000 and 2009 according to observations and simulations^27^.

The most important physical mechanism linking the Middle East dust with the ISM system in the above studies is the atmospheric heating caused by dust absorption of solar radiation, the efficiency of which is determined by the dust imaginary refractive index (IRI) in climate models. However, the dust IRI values used in these models are highly variable due to the lack of observational data of mineral dust compositions^28^. This variability may lead to unrealistic estimations of the dust impact on ISM rainfall. Dust single scattering albedo (SSA) showed an ability to influence the dust-induced ISM rainfall changes in a sensitivity modeling study^27^, but without comparing the modeled dust absorption properties with observations, it is hard to quantify the magnitude of the ISM rainfall response to dust aerosols. Moreover, this study did not include the whole Arabian Peninsula and might not be representative of the dust source regions in the Middle East. Therefore, in our study we performed numerical experiments in a larger domain to quantify the sensitivity of the ISM system to dust IRI in the Middle East and South Asia by employing various IRI data sets from previous literature. More importantly, we compared the modeled ISM system responses to dust aerosols with a composite analysis of ground-based observations, satellite retrievals, and atmospheric reanalysis data sets.

## Results

### Aerosol optical properties versus dust IRI

The sensitivity of the ISM system to dust absorption properties is investigated by employing the Weather Research and Forecasting model coupled with online chemistry (WRF-Chem). WRF-Chem has been shown to be a robust tool to study aerosol–climate interactions^25^,^29^,^30^. The model is integrated for June-July-August (JJA) 2008 with initial and boundary conditions from the European Centre for Medium-Range Weather Forecasts Interim Reanalysis (ERA-I) using prescribed sea surface temperature. A control experiment (CTRL) without dust is compared with six additional sensitivity experiments with dust for various dust IRI values (Table S1). Each of the seven experiments consists of 16 ensemble simulations generated by selecting various combinations of options for some chemical and physical processes in WRF-Chem, which include 4 aerosol-mixing rules, 2 shortwave radiation schemes, and 2 planetary boundary layer schemes (4 × 2 × 2 = 16). Please see Table S3 for details of the ensemble members. The results are shown as ensemble means. The differences between CTRL and sensitivity experiments represent the dust impacts, and the differences among sensitivity experiments indicate the impact of various dust IRI values.

**Figure 1:**
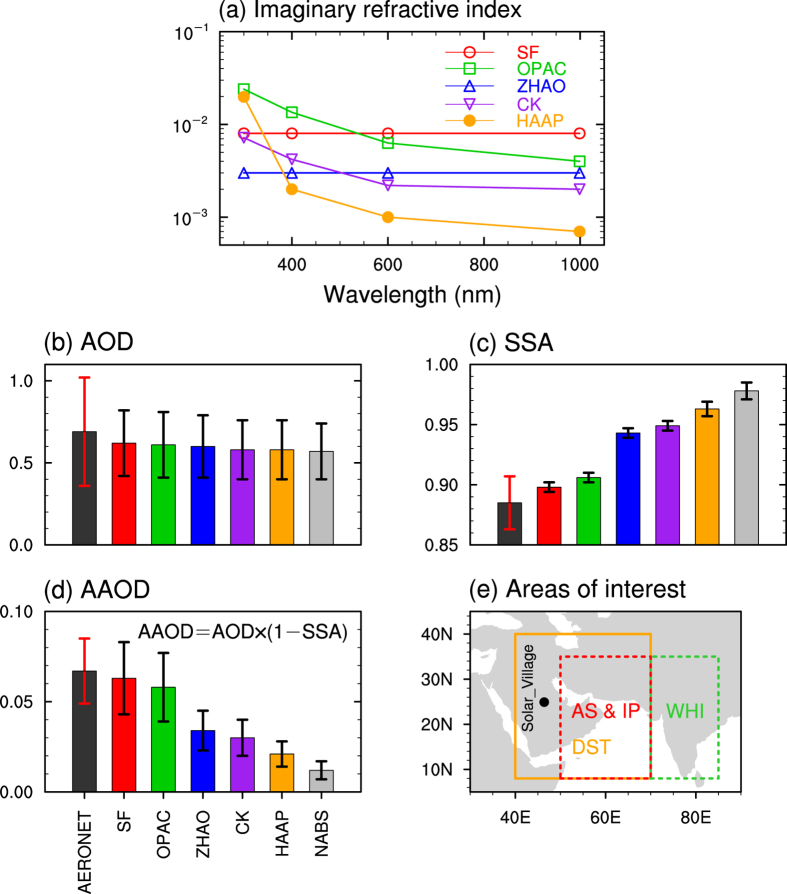
(a) Dust imaginary refractive indices at four wavelengths (300, 400, 600, and 999 nm) used in WRF-Chem. These data are extracted from various references and summarized in Table S1. Note that the real parts of dust refractive indices are 1.55 at the four wavelengths in all simulations. (**b–d**) Comparison of aerosol optical properties AOD, SSA, and AAOD, respectively, at Solar Village (marked by the black dot in Fig. 1e) in AERONET observations and the six disturbed WRF-Chem experiments for JJA 2008. The error bar on each bar represents one standard deviation. **(e)** Areas of interest in the study domain. The orange box (referred to as DST region) includes most of dust sources in the Middle East. The red and green boxes represent the IP and AS region and the whole Indian region, respectively. The Figure was created using NCAR (the National Center for Atmospheric Research) Command Language (NCL) of version 6.2.1 (http://dx.doi.org/10.5065/D6WD3XH5).

There is a large disparity in climate models to represent the dust IRI as a function of wavelength (Fig. 1a). Some models use decreasing values of dust IRI as wavelength increases from the ultraviolet to the infrared spectrum, while other models employ constant values. Hereafter, we will focus on dust IRI at 600 nm because solar irradiance is largest at this wavelength and therefore, differences in dust IRI will have the greatest potential impact. There is high variability in the dust IRI values used in various models, ranging from 0.001 to 0.008 at 600 nm.

Figure 1b–d respectively illustrate the modeled versus observed aerosol optical depth (AOD), SSA, and aerosol absorption optical depth (AAOD) at one Aerosol Robotic Network (AERONET) site (i.e., Solar Village). For all six sensitivity experiments, modeled AOD is very close to observed, with values of approximately 0.63 and 0.68, respectively. The simulated AOD decreases with the dust IRI due to decreases in the absorption of solar radiation by dust aerosols. The error bars in Fig. 1b–d represent the standard deviation based on the 16 ensemble members in each set of simulations. The ensemble spread represents the uncertainties in the model parameterizations of various physical and chemical processes. As discussed in one of our previous studies^25^, the biggest uncertainty is from the PBL scheme that controls the vertical diffusion of dust particles, followed by the shortwave radiation scheme. Figure 1c shows an observed SSA of 0.885 while simulated SSA ranges from 0.898 to 0.978. Of the six sensitivity experiments, SF and OPAC are more consistent with the observed SSA than the other models, with values of about 0.898 and 0.905, respectively. A previous study shows that using the OPAC dust IRI tends to overestimate the absorptive ability of dust aerosols in North Africa^31^; however, even with IRI values higher than OPAC, our simulations produce a lower AAOD compared to observations in the Middle East. This discrepancy could be attributed to a difference in the mineral composition of dust particles between the two regions. The iron composition is known to determine the absorptive ability of dust aerosols, and the dust particles in the Middle East contain more iron compounds (e.g., hematite, limonite, and magnetite) than those in North Africa^32^,^33^. As expected, the simulated dust AAOD in SF and OPAC are much larger than those in other sensitivity experiments are and more consistent with the observed values, as shown in Fig. 1d. Note that there is a positive feedback among dust emission, dust-heating effect in the atmosphere, and circulations over the Arabian Sea and Arabian Peninsula, which may contribute to the differences in AOD, SSA, and AAOD among various experiments^24^,^25^. Given the magnitude of dust-induced increase in wind velocity (0.5–1.0 m s^−1^) over dust source region (i.e. the Arabian Peninsula), which is about 10% to 20% of the absolute value, such an increase in wind velocity would result in 0.1^3^ to 0.2^3^ (0.1–0.8%) increase in dust emissions according to Eq. (1.1) in the Supplementary Information. Such a small increase in dust emissions is also seen in Fig. 1b, which shows that the AOD for different experiments are similar. Therefore, dust differences in dust optical properties among various experiments are mainly attributed to various dust IRI values.

### Dust radiative effects

**Figure 2:**
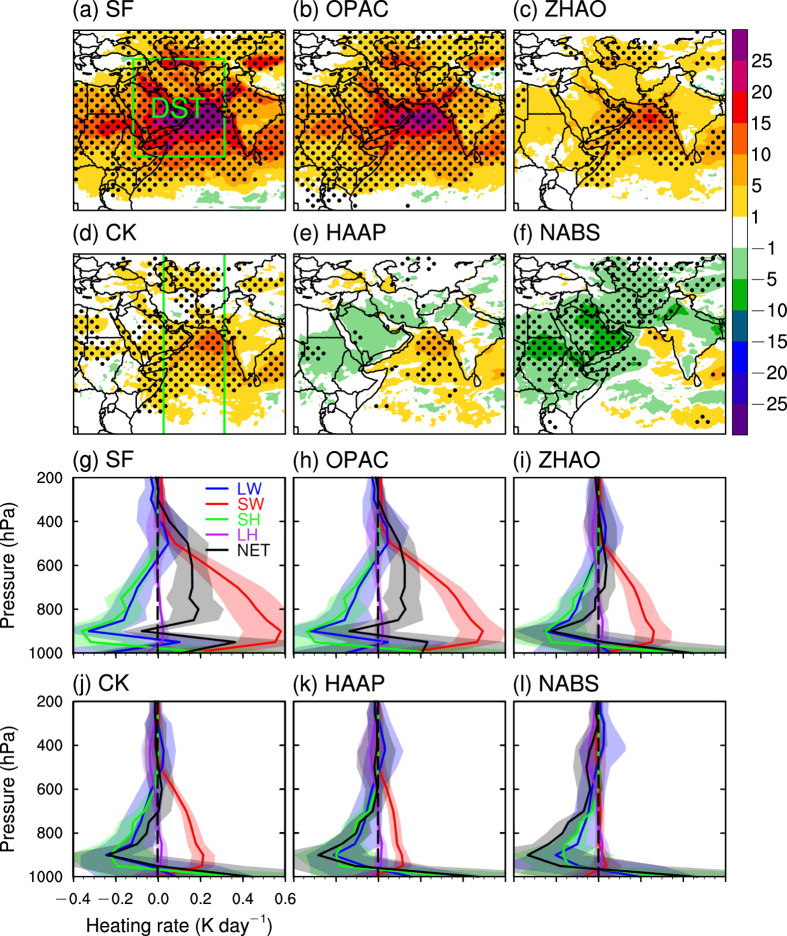
(**a–f**): Spatial patterns of the WRF-Chem ensemble means of the dust-induced net radiative effect (W m^−2^) in the atmosphere at all-sky conditions in the six disturbed experiments averaged for JJA 2008. Theblack dots represent grid points that are above the 95% confidence level based on a one-sided Student’s *t*-test. (**g**,**l**): Vertical profiles of the WRF-Chem ensemble means of the dust-induced atmospheric heating rate (K day^−1^) at all-sky conditions over the DST region averaged for JJA 2008. The green vertical lines in Fig. 2d represent the longitudes, between which the zonally means of atmospheric temperature and winds are calculated, as shown in Fig. 3. The shadings represent the differences that are above the 95% confidence level based on the ensemble spread for each heating source. The figure was created using NCL 6.2.1 (http://dx.doi.org/10.5065/D6WD3XH5).

Figure 2a–f demonstrate the spatial patterns of the dust net radiative effects in the atmosphere at all-sky conditions for JJA 2008. A similar plot at the clear-sky conditions is also produced for comparison (Figure S1 in the Supplementary Information). Dust net radiative effects in the atmosphere at all-sky conditions are generally consistent with those at clear-sky conditions, but with a slightly greater magnitude due to the cloud effects. Our analysis is focused on the all-sky conditions. Figure 2a shows that the strongest heating effects are mainly located over the AS and south AP with a magnitude of about 25–30 W m^−2^. As the absorptive ability of dust particles becomes weaker and ultimately is reduced to zero, the heating effects become weaker (Fig. 2a–d) and ultimately change to cooling effects (Fig. 2e,f). The magnitude of 5–10 W m^−2^ of the cooling effects (Fig. 2f) is much smaller than the strong heating effects (Fig. 2a,b). The net cooling effects in Fig. 2e,f are due to the reemitting of long-wave radiation from the ground by the dust layers. Note that the dust-induced radiative effects here include all sources (i.e. direct, indirect, and semi-direct radiative effects). Generally the magnitude of aerosol’s indirect and semi-direct effects is much smaller than its direct effect^34^,^35^.

Figure 2g–l show the area-averaged vertical profiles of dust-induced heating rates of the atmosphere from various sources in the DST region (the orange box in Fig. 1e). In Fig. 2g, the maximum heating (0.6 K s^−1^) and cooling (0.35 K s^−1^) effects are located at 900 hPa, due to shortwave radiative effect and the combined effects of long-wave radiative and sensible heating, respectively. The latent heating effect is very small. The net heating effect, defined as the overall average of shortwave and long-wave radiative effects along with sensible and latent heating effects, has a positive value of about 0.2 K s^−1^ extending from 850 to 500 hPa. As the absorptive ability of dust aerosols is reduced to zero (Fig. 2g–l), both heating and cooling rates decrease in magnitude. However, the heating rate decreases more quickly and the net effect shifts from heating to cooling. The shadings in Fig. 2g–l represent the 95% confidence interval based on the ensemble spread. Moreover, the dust-induced heating in the lower to mid-troposphere (Fig. 2g,h) can destabilize the troposphere, thereby benefitting the initialization and development of the convection system in the Indian monsoon region, as discussed in the following paragraph.

### Dust-induced changes in monsoon circulation and water vapor transport

**Figure 3:**
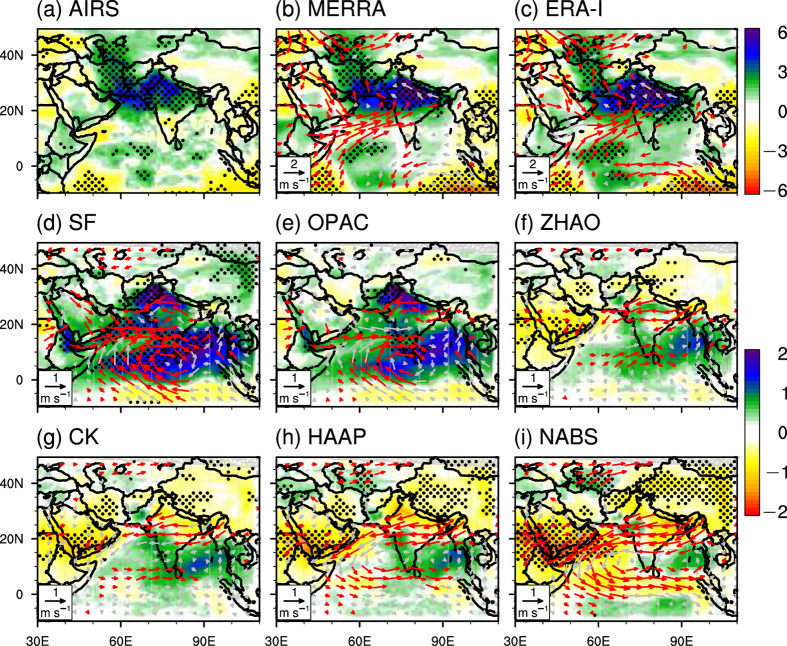
(**a**–**c**) The spatial patterns of differences in precipitable water (shadings; unit: mm) and winds (arrows; units: mm s^−1^) at 850 hPa based on a composite analysis method. The composite months are selected as 12 months with the highest AOD anomalies (in the order of AOD anomalies from low to high: 2011/08, 2012/06, 2008/07, 2009/07, 2006/07, 2007/06, 2009/08, 2003/07, 2013/06, 2011/07, 2011/06, and 2008/06) and 12 months with the lowest AOD anomalies (in the order of AOD anomalies from low to high: 2006/06, 2005/06, 2004/07, 2007/08, 2014/07, 2009/06, 2005/07, 2010/06, 2006/08, 2010/07, and 2014/06) of 36 months in 2003–2014. The AOD is from MISR monthly data set and area-averaged over the Iranian Plateau and the Arabian Sea (marked by the red box in Fig. 1e). (**d–i**) The spatial of the dust-induced changes in precipitable water and winds in the six model experiments. The black points indicate the grids that are above the 95% confidence level. The red arrows in Fig. 3b–i are above the 95% confidence level. The figure was created using NCL 6.2.1 (http://dx.doi.org/10.5065/D6WD3XH5).

Figure 3 shows the spatial patterns of dust-induced changes in horizontal winds and precipitable water based on satellite retrieval, reanalysis data and model results. Figure 3a–c show the results from a composite analysis. The JJA months over a 12-year period (2003–2014) were sorted according to MISR monthly AOD values area-averaged over the Arabian Sea and the Iranian Plateau (marked by the red box in Fig. 1e). The averaged values for relevant meteorological variables within two groups composed of the 12 most and least dusty months were compared. The El Niño Southern Oscillation (ENSO) has been shown to have great impacts on the South Asia climate^2^,^10^,^36^,^37^,^38^,^39^. In order to evaluate the effect of ENSO in the composite analysis, the scatter plot of the normalized AOD versus Niño 3.4 index is produced. Figure S2 shows that in the 7 La Niña months, 2 months have abnormal low AOD and 3 months have abnormal high AOD; similarly, in the 8 El Niño months, 2 months have abnormal low AOD and 2 months have abnormal high AOD. There is no evidence that the AOD anomalies in the Arabian Sea and the Arabian Peninsula tend to occur in ENSO month.

Figures 3a–c illustrate that the atmospheric heating effect over the Arabian Sea and Iranian Plateau results in a convergence anomaly at 850 hPa over the northern Arabian Sea in both MERRA and ERA-I reanalysis data with a magnitude of 2 m s^−1^. The southwesterly winds of the convergence anomaly transport more water vapor from the Arabian Sea to the Indian subcontinent, resulting in roughly a 6 mm increase in precipitable water in the area of Pakistan and India (Fig. 3a–c) based on satellite and reanalysis data. The spatial patterns of the simulated changes in monsoon circulations and precipitable water are very similar to satellite and reanalysis composite results, but with a smaller magnitude of about 1 m s^−1^ and 2 mm, respectively (Fig. 3d). The dust-induced convergence and positive precipitable water anomalies become weaker (Fig. 3d,e) and change to divergence and negative anomalies (Fig. 3f–i) as dust aerosols become less absorptive. The changes in vertical profiles of atmospheric circulation and temperature are also analyzed and shown in Figure S3. It is worth pointing out that the dust-induced increase in rainfall can result in additional latent heat release, which may strengthen the monsoon circulation. This constitutes another positive feedback in addition to the wind and dust emission feedback discussed above.

### Sensitivity of monsoon rainfall changes to dust IRI

**Figure 4:**
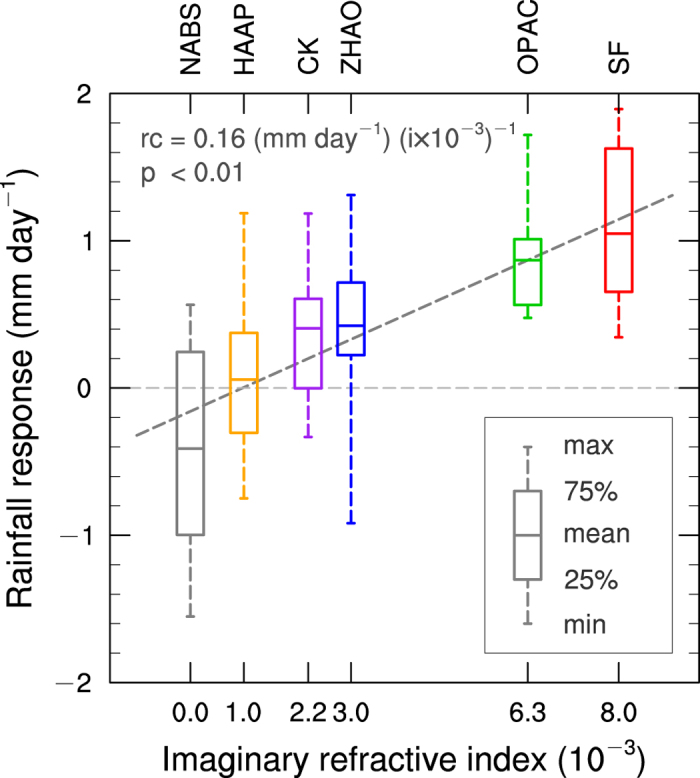
The response of the WRF-Chem simulated rainfall (mm day^−1^) in India (8–35°N, 70–85°E) to dust aerosols with different dust imaginary refractive indices. Simulations are for JJA 2008. The slope dash line represents the linear trend of the means of rainfall responses versus dust IRI. The figure was created using NCL 6.2.1 (http://dx.doi.org/10.5065/D6WD3XH5).

The dust-induced ISM rainfall changes are area-averaged in India and northern Pakistan (see the green box in Fig. 1e) for JJA 2008 and shown in Fig. 4 versus the dust IRI. The averages of the dust-induced positive ISM rainfall anomalies decrease from 1 to 0.05 mm day^−1^ as dust aerosols become less absorptive. When dust is non-absorptive, the rainfall response is negative (−0.4 mm day^−1^). Figure S4(d,e) show that the modeled strongest rainfall increases occur in Pakistan, northern India, and southwest coastal India, which is generally^19^,^26^ consistent with our observational study^24^ and the composite analysis (Figure S4(a–c)), except for a non-significant rainfall decrease in the model but a significant rainfall increase in observations in East-Central India.

## Discussion

Using a high-resolution regional climate model coupled with online chemistry, satellite retrievals, and reanalysis data sets, we examined the sensitivity of the ISM rainfall to dust absorption properties. Modeling results show that both the magnitude and direction of dust-induced ISM rainfall changes are highly sensitive to dust absorptive properties; dust aerosols with stronger absorption can result in larger increases in the ISM rainfall. This finding is generally consistent with a previous study that absorptive aerosols result in more ISM rainfall than scattering aerosols^27^. Comparing the model results for radiation, temperature, circulation, and precipitable water with results from composite analysis based on satellite and reanalysis data sets indicates that all model experiments significantly underestimate the magnitude of dust-induced changes. Moreover, the scatter plot between normalized AOD and Niño 3.4 Index shows that ENSO has little effect on Middle East dust emissions in the boreal summer. Our findings address the importance of dust absorptive properties in modulating the ISM rainfall variability on the intra-seasonal time scale and imply a necessity of developing a parameterization for dust IRI (e.g. using the global map of soil mineral compositions and its relationship with dust absorptive ability) to replace the globally constant values in the current climate models.

## Methods and Data Sets

### Model description

This study employs the Weather Research and Forecasting model coupled with online chemistry (WRF-Chem, V3.5). Aerosol dynamics including particle formation, condensational growth, coagulation, and deposition are modeled using the Modal Aerosol Dynamics model for Europe (MADE) primary aerosol scheme coupling the Secondary Organic Aerosol Model (SORGAM) aerosol scheme. The dust emission is calculated by the Goddard Chemistry Aerosol Radiation and Transport (GOCART) scheme based on the prognostic wind speed 2 m above the surface and the erodibility map. The radiative feedback of dust aerosols is considered in various shortwave and long-wave radiation schemes. These model schemes have been described in detail in the Supplementary Information and previous studies^40^,^41^,^42^.

### Experimental design

Seven sets of experiments are designed to quantify the sensitivities of the ISM system to dust IRI uncertainties. A control set of experiments without dust emission is compared to six additional sets of experiments that include dust emission. The six sensitivity sets vary in dust IRI values, but are otherwise identical. The details of the dust IRI are listed in Table S1. Each set of experiments contains 16 ensemble simulations, which are created by using various options for the planetary boundary layer, SW radiation, and aerosol chemical mixing rules. Further details on the design of ensemble simulations can be found in a previous study^25^.

### Data sets

The Aerosol Robotic Network (AERONET) program is a global ground-based network established by NASA for measuring aerosol microphysical and optical properties. AERONET provides direct sun photometer products such as AOD, fine and coarse mode AOD, Angstrom parameter, as well as derived inversion products such as single scattering albedo (SSA), aerosol absorption optical depth (AAOD), and so on. In this study, AOD, SSA, and AAOD data sets during JJA 2008 at the site of Solar Village (shown as a black dot in Fig. 1e) are used to evaluate model performance in simulating aerosol optical properties. The duration of data collection at other sites in our study domain were too short (i.e. several days) for evaluating model performance.

The Multi-angle Imaging Spectroradiometer (MISR) onboard NASA’s Terra satellite is designed to observe detailed aerosol physical and optical properties at a global scale. MISR images the Earth’s atmosphere and surface with nine cameras in each of the four wavelengths (blue, green, red, and near-infrared), enabling it to retrieve highly accurate information about aerosol properties such as aerosol optical depth, particle size, shape, and composition. Unlike MODIS aerosol products, which are retrieved using “dark-target” and “deep-blue” algorithms over low- and high-reflect surfaces, respectively, MISR uses a single algorithm to retrieve aerosol properties over both land and ocean, providing more consistent aerosol products at a global scale. The scan swath of MISR is 360 km, resulting in a long global span time of 9 days. This study employs MISR monthly retrievals (level three, version 004) with a resolution of 0.5° × 0.5° (available form ftp://l5eil01.larc.nasa.gov/MISR/MIL3MAEN.004).

The Atmospheric Infrared Sounder (AIRS) aboard NASA’s Aqua satellite employs cutting-edge infrared technology to measure the Earth’s atmospheric temperature and water vapor profiles at a global scale. With 2378 spectral channels, AIRS has a much higher spectral resolution than prior instruments and provides much more accurate data for atmospheric temperature and water vapor. The scan swath of AIRS is roughly 1600 km, giving AIRS a global span time of 1 to 2 days. AIRS monthly standard physical retrievals (level three, version 006) is used with a resolution of 1° × 1° (available from ftp://acdisc.sci.gsfc.nasa.gov/ftp/data/s4pa/Aqua_AIRS_Level3/AIRS3STM.006/).

Atmospheric temperature, column integrated water vapor, and circulations from AIRS and model results are compared with those from the Modern Era-Retrospective Analysis for Research and Applications^43^ (MERRA) (1/2° × 2/3°) as well as ERA-I^44^ (0.5° × 0.5°). The Niño 3.4 index is from the Climate Prediction Center of the National Oceanic and Atmospheric Administration, which is available here: http://www.cpc.ncep.noaa.gov/data/indices/sstoi.indices.

## Additional Information

**How to cite this article**: Jin, Q. *et al*. High sensitivity of Indian summer monsoon to Middle East dust absorptive properties. *Sci. Rep.* **6**, 30690; doi: 10.1038/srep30690 (2016).

## Supplementary Material

Supplementary Information
