# Supplementary material for: High sensitivity of Indian summer monsoon to Middle East dust absorptive properties
Source: Sci Rep. 2016 Jul 28;6:30690. doi: 10.1038/srep30690 (PMC4964619; doi:10.1038/srep30690)
Supplement: Supplementary Information [file srep30690-s1.pdf]

Supplementary Information for

High sensitivity of Indian summer monsoon to Middle East dust absorption properties

Qinjian Jin[1, 2], Zong-Liang Yang[1,*], Jiangfeng Wei[1]

[1]Department of Geological Sciences, University of Texas at Austin, 1 University Station C1100, Austin, Texas, USA 78712

[2]Now at Department of Earth, Atmospheric, and Planetary Sciences, Massachusetts Institute of Technology, 77 Massachusetts Avenue, Cambridge, MA 02139

Correspondence to: Z.-L. Yang (liang@jsg.utexas.edu)

This Supplementary Information provides additional detailed information about model configuration, experimental design, and the model representation of dust emissions, aerosol mixing rules, and aeraosol optical properties.

1. Dust IRI values

The dust IRI values used in this study are listed in Table S1.

Table S1. List of experiments with dust imaginary refractive indices at four wavelengths (unit: nm) used in various climate models. The real part of the dust refractive index is set to 1.55 at these four wavelengths in all experiments. The dust IRI in RegCM4.5 is shown here for comparison purpose, but not used in our experiments.

Experiment	Model	Imaginary part				Reference
		300	400	600	999	
SF	libRadtran	0.008	0.008	0.008	0.008	[45,46]
OPAC	CESM-CAM	0.024	0.0135	0.0063	0.004	[47-49]
	RegCM4.5	0.0284	0.0154	0.0039	0.0031	[50]
ZHAO	WRF-Chem	0.003	0.003	0.003	0.003	[51,52]
CK	NASA GEOS-5	0.0072	0.0042	0.0022	0.002	[53-55]
HAAP	ECHAM-HAM	0.0198	0.002	0.001	0.0007	[56-59]
NABS	Not applicable	0	0	0	0	

libRadtran: library for radiative transfer; CESM-CAM: The Community Earth System Model-the Community Atmosphere Model; RegCM: REGional Climate Model; GEOS: The Goddard Earth Observing System Model; ECHAM5.5-HAM2: a global aerosol-climate model originated from ECMWF and developed in Hamburg, Germany.

2. Description of model configuration and ensemble experimental design.

The key physical and chemical schemes used in WRF-Chem experiments are listed in Table S2. For shortwave and PBL scheme, multiple options are used to create physical and chemical perturbed ensemble simulations, which are listed in Table S3.

Table S2. Configuration options of WRF-Chem used in this study.

Atmospheric Process		Model Option
Physics	Long-wave radiation	RRTMG
	Shortwave radiation	RRTMG or Goddard
	Surface layer	Monin-Obukhov
	Land surface	Noah
	Boundary layer	YSU or BouLac
	Cumulus clouds	Grell-Freitas
	Cloud microphysics	Lin et al.
Chemistry	Gas-phase chemistry	RADM2
	Aerosol chemistry	MOSAIC-SORGAM with aqueous reactions
	Photolysis	Fast-J

Emission	Dust emission	GOCART
	Sea-salt emission	MADE-SORGAM
	Anthropogenic emission	Reanalysis of the Tropospheric and Emission Database for Global Atmospheric Research
	Biogenic emission	The Model of Emissions of Gases and Aerosols from Nature version 2
	Fire emission	MODIS

YSU and BouLac stand for the Yonsei University and Bougeault–Lacarrère planetary boundary layers, respectively. RADM2 is the second-generation Regional Acid Deposition Model gas-phase chemical mechanism.

The 16 ensemble simulations in the control and each of the six sensitivity experiments are created by selecting various schemes for physical and chemical processes to take into account the potential model uncertainties. These processes include the planetary boundary layer (2 options), aerosol chemical mixing rules (4 options), and shortwave radiation (2 options) and the details on the options for these processes are listed in Table S2. The reason for addressing these three processes is that they largely determine the radiative impacts of dust aerosols on the climate system.

Table S3. Various physical and chemical schemes employed to create the 16 ensemble members.

Scheme	Option	Description
Shortwave radiation	Goddard	1. 11 spectral bands (7 UV, 1 PAR, 3 infrared),<br>2. A two-stream adding algorithm<br>3. No interacts with sub-grid clouds
	RRTMG	1. 14 spectral bands (3 UV, 2 PAR, 9 infrared),<br>2. A two-stream algorithm for multiple scattering<br>3. Represents sub-grid cloud variability by McICA with maximum-random cloud overlap.
Planetary boundary layer	YSU	1. First-order closure<br>2. *K* profile and non-local mixing<br>3. Explicit treatment of entrainment
	BouLac	1. One-and-a-half order closure<br>2. Prognostic turbulent kinetic energy equation
Aerosol chemical mixing rules	Volume	1. Internal-mixing of aerosol composition<br>2. Full Mie calculations are called only at the first time step
	Maxwell	1. Randomly distributes black carbon within a particle<br>2. Full Mie calculations are called only at the first time step
	Exact volume	Same as volume, but use full Mie calculation at each time step
	Exact Maxwell	Same as Maxwell, but use full Mie calculation at each time step

UV, PAR, and McICA stand for ultraviolet, photosynthetically active radiation, and the Monte

Carlo Independent Column Approximation, respectively. RRTMG is the Rapid Radiative Transfer Model GCM application.

3. Dust net radiative effect

The dust-induced net radiative effects and heating rates in the atmosphere at clear-sky conditions, except for heating rates of sensible and latent heat at all-sky conditions, because WRF-Chem calculates heating rates of sensible and latent only at all-sky conditions.

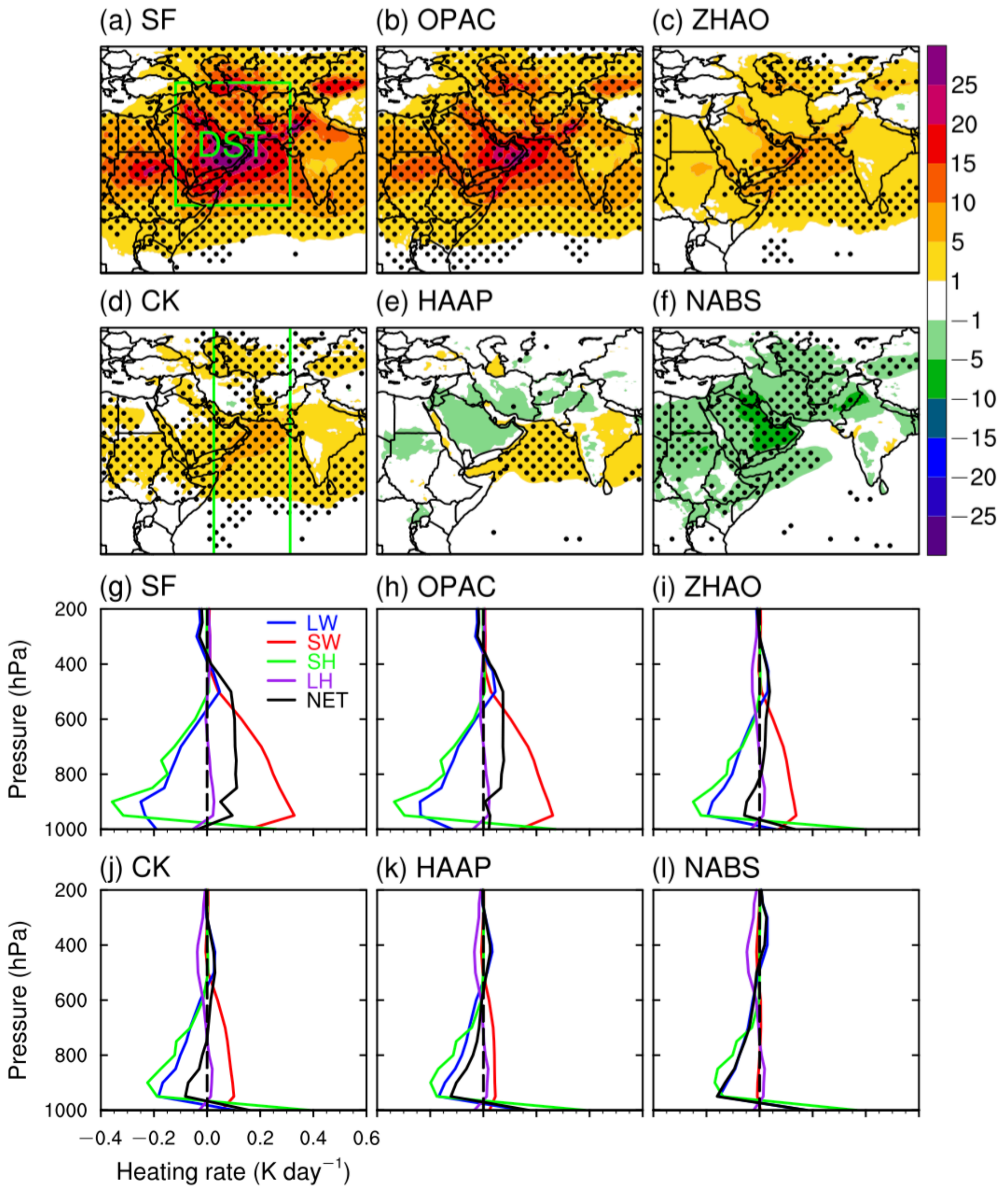

Figure S1. (a)–(f): dust net radiative effects (W m^{-2}) in the atmosphere at clear-sky conditions. (g)–(l) dust-induced heating rates (K day^{-1}) at clear-sky conditions for shortwave and long-wave and net effect, and at all-sky conditions for sensible and latent heat. The figure was created using NCAR (the National Center for Atmospheric Research) Command Language (NCL) of version 6.2.1 (http://dx.doi.org/10.5065/D6WD3XH5).

4. Relationship between AOD and ENSO

The relationship between AOD over the Arabian Sea and the Arabian Peninsula and ENSO is represented by the scatter plot of normalized AOD versus Niño 3.4 index, as shown in Figure S2. Based on Figure S2, AOD anomalies can occur in both La Niña and El Niño months. No solid evidence was found for a close relationship between AOD and ENSO in our analysis.

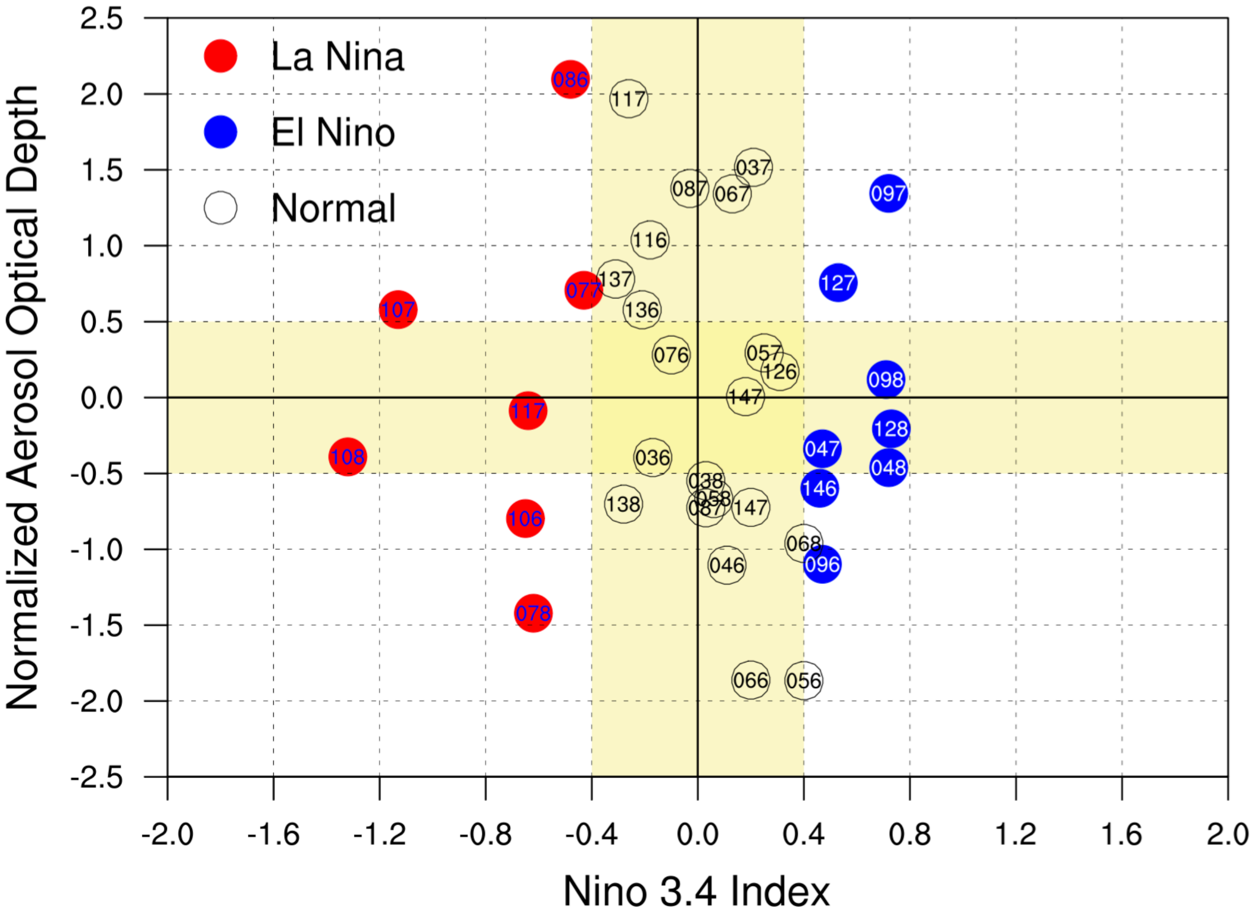

Figure S2. Scatter plot of normalized AOD and Niño 3.4 index during JJA 2003–2014. The AOD is area-averaged over the Arabian Sea and the Arabian Peninsula as marked by the red box in Figure 1e. The Niño 3.4 index is from the Climate Prediction Center of the National Oceanic and Atmospheric Administration. The first two digits of the three-digit numbers in the circles represent the last two digits of the year from 2003 to 2014, and the last digit represents the month from June to August. The figure was created using NCAR (the National Center for Atmospheric Research) Command Language (NCL) of version 6.2.1 (http://dx.doi.org/10.5065/D6WD3XH5).

5. Dust-induced changes in vertical profiles of atmospheric temperature and circulation.

Figure S4a, based on Atmospheric Infrared Sounder (AIRS) satellite data, exhibits strong atmospheric warming effects within 900–600 hPa and 10°–25° N over the Arabian Sea and 900–300 hPa and 30°–35° N over the Iranian Plateau, with magnitudes of 1.0 and 1.5 K, respectively. The consistent heating patterns are also seen in the Modern Era-Retrospective Analysis for Research and Applications (MERRA) and ERA-I reanalysis data (Figures S4b and S4c). Due to atmospheric heating over the Arabian Sea, a southerly wind anomaly is observed within 900–800 hPa and 5°–20° N as well as an upward wind anomaly within 900–500 hPa at around 20° N (Figures S2b and S2c). Another important circulation change is the upward wind anomaly from the surface to the upper troposphere over the Iranian Plateau.

The simulated changes in atmospheric temperature show positive anomalies in the lower- to mid-troposphere over the Arabian Sea as well as in the upper troposphere over the Iranian Plateau (Figure S4d), which are similar to satellite and reanalysis data, but with a smaller magnitude (0.5 K). Due to the positive temperature anomalies, upward wind anomalies are simulated over the Arabian Sea (Figure S4d), which are similar to the observed wind anomalies (Figures S4b and S4c). However, no significant wind change is simulated over the Iranian Plateau, which is probably due to the underestimation of atmospheric heating effects by the model in this area. As dust aerosols become less absorptive, the positive temperature anomalies become weaker in (Figures S4d and S4e) and eventually negative in Figures 3f–3i. At the same time, the upward wind anomalies weaken (Figures S4d–S4i).

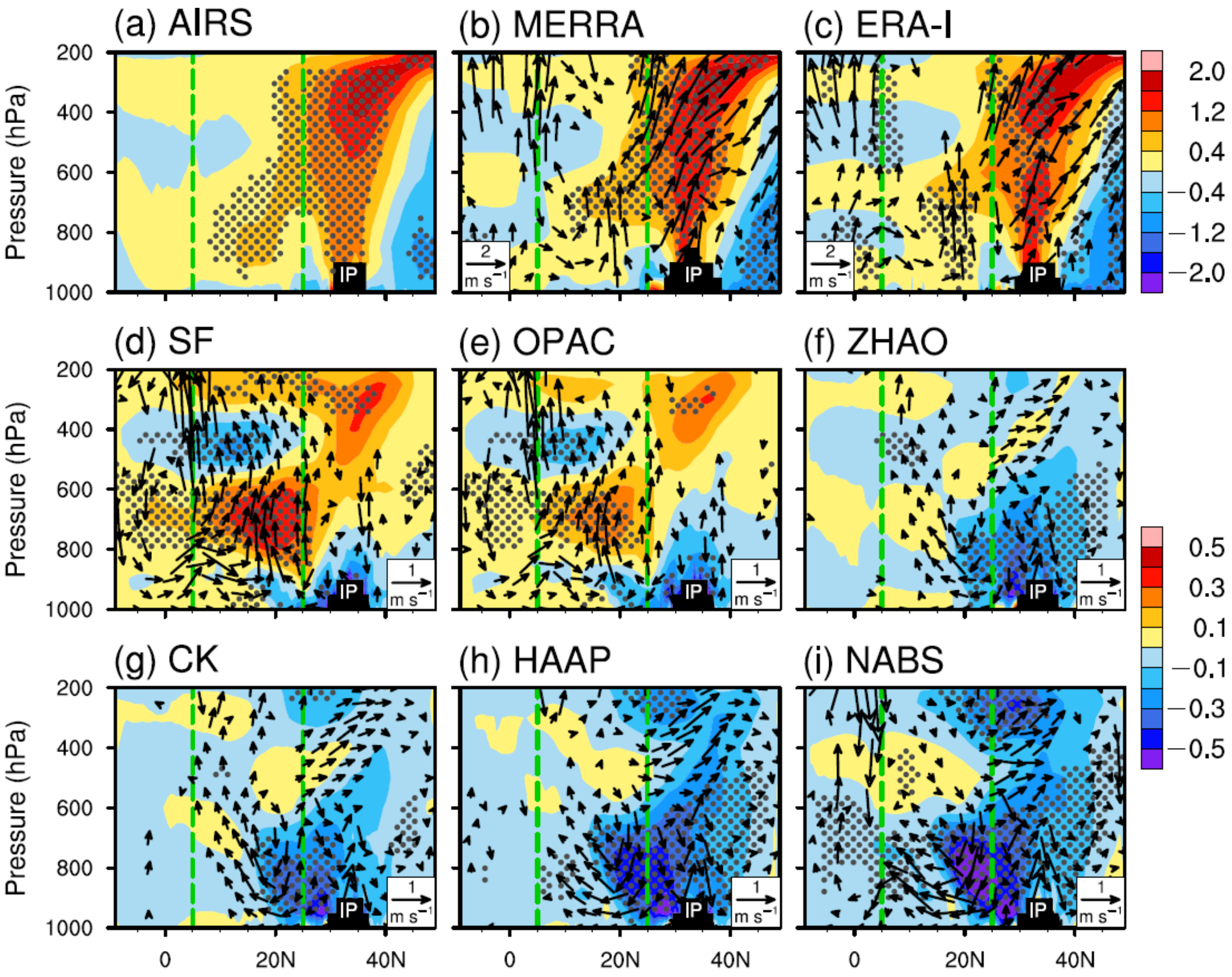

Figure S3. (a)–(c) The zonally (i.e., 50°–70° E) averaged changes in atmospheric temperature (shadings; unit: K) and winds (arrows; units: m s^{-1}) against the pressure level for JJA 2008 based on the same composite analysis method as in Figure 3. **(d)–(i)** Dust-induced changes in atmospheric temperature and winds in the six model experiments. The grey dots represent differences above the 95 % confidence level. All wind vectors shown are above the 95 % confidence level. The vertical wind are scaled by factors of 10 and 800 in reanalysis and model results, respectively. The figure was created using NCL 6.2.1 (http://dx.doi.org/10.5065/D6WD3XH5).

6. Modeled rainfall response to dust

Figure S3 shows the rainfall responses in India to dust aerosols over the Arabian Sea and the Arabian Peninsula, which is discussed in the part of “Sensitivity of Monsoon rainfall changes to dust IRI” in the main text.

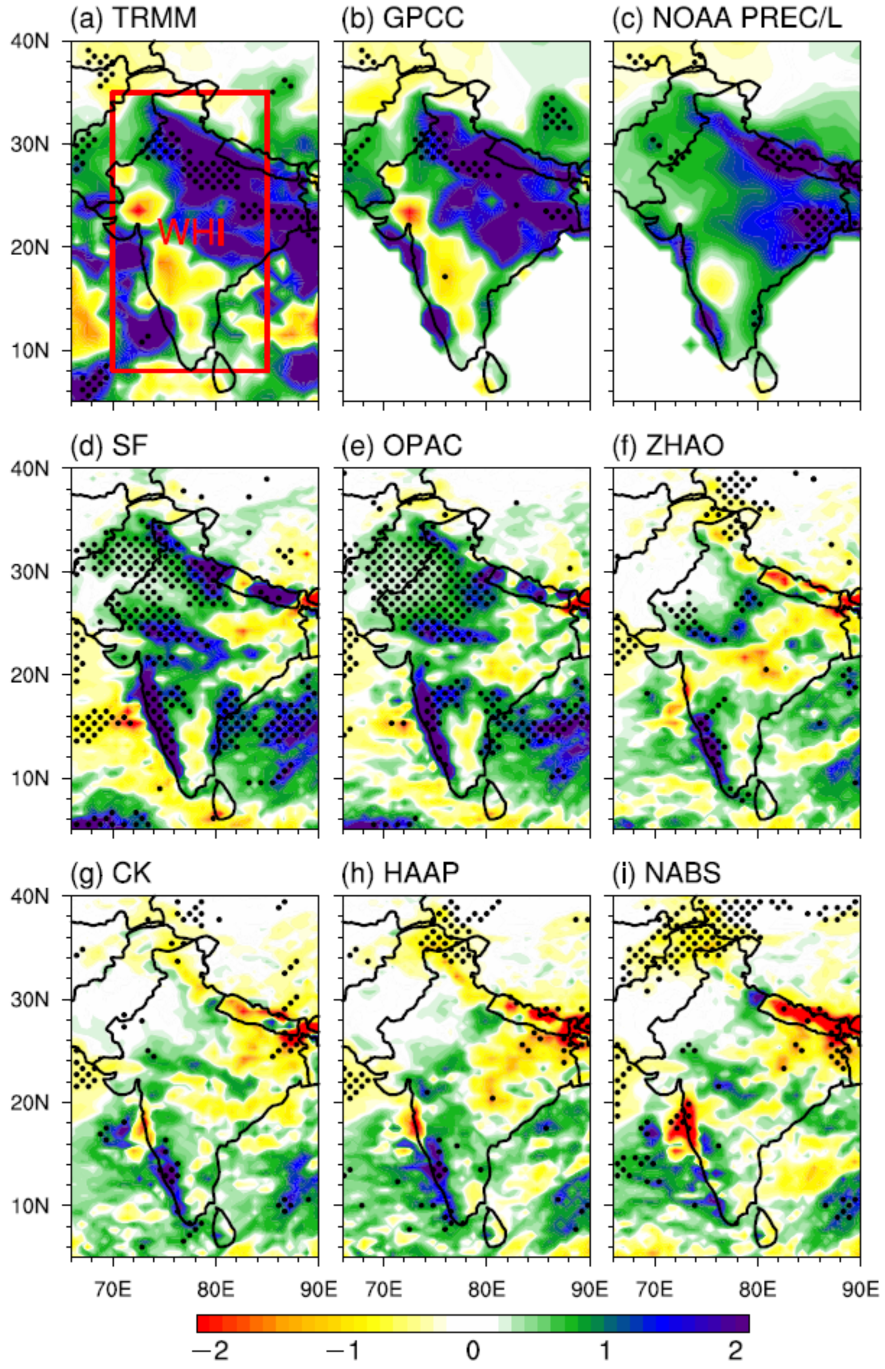

Figure S4. Spatial patterns of (a)–(c) rainfall differences (mm day^{-1}) from three observations based on the same composite analysis method as in Figure 3 and **(e)–(i)** the WRF-Chem ensemble means of the total rainfall (i.e. sum of stratiform and convective rainfall) responses (mm day^{-1}) to dust aerosols in 16 members averaged for JJA 2008. Rainfall responses are calculated by subtracting rainfall in the control experiment without dust from rainfall in the sensitivity experiments with dust at various dust IRI values. The black dots represent grid points that have a 90 % confidence level based on a one-sided Student's *t*-test. The figure was created using NCAR (the National Center for Atmospheric Research) Command Language (NCL) of version 6.2.1 (http://dx.doi.org/10.5065/D6WD3XH5).

7. Representation of dust emissions and optical properties in WRF-Chem

7.1. Dust emission

Dust emission is calculated by the Goddard Chemistry Aerosol Radiation and Transport model following Eq. (1.1) (Ginoux et al., 2001).

$$G_i = CF_iE_iu_{10}^2\left(u_{10} - u_{th,i}\right) \quad (1.1)$$

where G_i is the vertical dust emission flux (Kg m^{-2} s^{-1}), the subscript i represents dust five size bin, C (Kg m^{-3} s^{2}) is the dust emission tuning factor, currently it is 0.85×10^{-8}, F_i is a fraction of each size bin of dust in emission, u_{10} is the wind velocity at 10 m above the ground, E_i is the map of soil topographic erodible factor, $u_{th,i}$ is the threshold of wind velocity for dust emission under which dust emission is prevented. The threshold of wind velocity is calculated following Eq. (1.2)–(1.4).

$$u_{th0,i} = 0.0013\sqrt{\frac{\rho_{d,i}gR_{d,i}}{\rho_{air}}\left(1+\frac{0.006}{\rho_{d,i}gR^{2.5}{}_{d,i}}\right)}\Bigg/\sqrt{1.928\times\left(1331R_{d,i}{}^{1.56}+0.38\right)^{0.092}-1} \quad (1.2)$$

$$f\left(w\right) = 1.2 + 0.2\log_{10}\left[\max\left(10^{-3}, w\right)\right] \quad (1.3)$$

$$u_{th,i} = u_{th0,i}f(w) \quad (1.4)$$

In Eq. (1.2), $\rho_{d,i}$ is the density of dust aerosols, g is the gravitational accelerate velocity, $R_{d,i}$ is the averaged radius of dust aerosols, and ρ_{air} is the density of the air. In Eq. (1.3), $f(w)$ represents the influence of soil moisture on the threshold of wind speed for dust emission, where w is defined by the volumetric soil moisture over soil porosity.

The mass of dust emission is then summed up over the five bins to get the total dust emission. Then 93% of mass of the total dust emission is assigned to the coarse mode (i.e. $M_{dust,coarse}$ in Eq. (2.1)), and the remaining 7% goes into the accumulation mode. The 93% and 7% of the total emitted dust mass is also converted to the number concentrations and assigned to the coarse (N_{coarse}) and accumulation (N_{acc}) modes, respectively, assuming the number mean size and standard deviation of 0.3 μm and 1.7 for the accumulation mode and 6.0 μm and 2.2 for the coarse mode. The Hatch–Choate conversion equation for mean size is used for this conversion between mass and number mean sizes.

Table 1. The size dependent parameters used in the GOCCART dust emission schemes, including the mean radius, lower, and upper radius boundaries of dust aerosols, the density of dust aerosols, and the fraction of each size bin of dust in emission.

Bin #	$R_{d,i}$ (μm)	$R_{low,i}$ (μm)	$R_{up,i}$ (μm)	$\rho_{d,i}$ (Kg m^{-3})	F_i
1	0.73	0.1	1.0	2500	0.10
2	1.4	1.0	1.8	2650	0.25
3	2.4	1.8	3.0	2650	0.25
4	4.5	3.0	6.0	2650	0.25
5	8.0	6.0	10	2650	0.25

The distribution of aerosol size in WRF-Chem is represented in sectional (bin) and modal (mode) methods. The modal method, which includes three overlapping modal modes: Aitken (0.01–0.1 μm), accumulation (0.1–1.0 μm), and coarse (1.0–10.0 μm), is employed in MODA-SORGAM aerosol scheme, assuming a lognormal distribution within each mode, as formulated in Eq (2.1).

$$n_m\left(D_m;\bar{D}_m,\sigma_m\right)=\frac{N_m}{\sqrt{2\pi}\ln\sigma_m}\exp\left[-\frac{\left(\ln D_m-\ln\bar{D}_m\right)^2}{2\ln^2\sigma_m}\right] \tag{2.1}$$

where, N_m and σ_m are respectively aerosol number concentration and standard deviation of aerosol size distribution. Aerosol mass ($M_{s,m}$) and number concentration (N_m) are predicted based on aerosol dynamical processes, which includes emission, nucleation, condensation, coagulation, dry deposition, and wet deposition. The subscript "s" stands for the eight species in the mixture of aerosols, including sulfate, nitrate, ammonium, black carbon (BC), organic carbon (OC), dust, sea salt, and water; the subscript "m" stands for the three modal modes. The standard deviation of aerosol size σ_m is currently assumed to be 1.7, 2.0, and 2.5 at Aitken, accumulation, and coarse modes, respectively. Note that OC includes nine aerosol species.

The number averaged radius ($\bar{D}_m$) of aerosols at a specific mode is estimated using Eq. (2.2) and (2.3):

$$V_m=\sum_{s=1}^{7}M_{s,m}/\rho_s \tag{2.2}$$

$$\bar{D}_m=\left(\frac{\pi}{6}\frac{V_m}{N_m}\right)^{1/3} \tag{2.3}$$

where V_m and ρ_s are the volume of all aerosol species at a specific mode and the density of a specific aerosol species. Water is not included in the summation of Eq. (2.2), because the size bins are defined by aerosol dry radius.

Aerosol mass and number from modal distribution is divided into individual sections or bins before passed into the Mie calculation. Currently the model use the same eight size bins as default MOSAIC aerosol scheme. The lower and upper boundaries of dry-diameter of aerosols are listed in Table 2.

Table 2. Particle dry-diameter (μm) ranges and means for eight size bins employed in MIE code for aerosol optical properties.

Bin #	Lower Diameter ($D_{low,b}$)	Upper Diameter ($D_{up,b}$)	Mean Diameter
1	0.0390625(=10/2^8)	0.078125(=10/2^7)	0.05859375(=15/2^8)
2	0.078125	0.15625	0.1171875
3	0.15625	0.3125	0.234375
4	0.3125	0.625	0.46875
5	0.625	1.25	0.9375
6	1.25	2.5	1.875
7	2.5	5	3.75
8	5	10	7.5

7.3. The conversion from modal to sectional representation

The conversion of aerosol size distribution from modal to sectional representation is conducted using the *Q*-function. $Q(x)$ is the probability that a normal random variable will have a larger value by x standard deviation than the mean. Given a known lognormal distribution, the probability that a random variable falls between the lower boundary of radius, $D_{low,b}$, and the upper boundary of radius, $D_{up,b}$, is calculated by Eq. (3.1). Based on the characteristic of the lognormal distribution that the shape of this distribution is the same for all moments. In other words, if the number distribution is lognormal, the surface and mass distribution is also lognormal. However, the mass mean/median radius is different from the number mean/median radius. The median sizes of various order moments can be determined from a known distribution using the Hatch–Choate equation (Eq. (3.2)). For mass distribution, the order, k, equals three.

$$F_{m,b}^{N} = \frac{1}{2}\left[Q\left(\frac{\ln D_{low,b} - \ln \bar{D}_m}{\sqrt{2}\ln\sigma_m}\right) - Q\left(\frac{\ln D_{up,b} - \ln \bar{D}_m}{\sqrt{2}\ln\sigma_m}\right)\right] \tag{3.1}$$

$$\ln \bar{D}^k = \ln \bar{D} + k\left(\ln\delta\right)^2 \tag{3.2}$$

$$F_{m,b}^{M} = \frac{1}{2}\left\{ Q\left[\frac{\ln D_{low,b} - \left(\ln \bar{D}_m + 3\left(\ln\sigma_m\right)^2\right)}{\sqrt{2}\ln\sigma_m}\right] - Q\left[\frac{\ln D_{up,b} - \left(\ln \bar{D}_m + 3\left(\ln\sigma_m\right)^2\right)}{\sqrt{2}\ln\sigma_m}\right]\right\} \quad (3.3)$$

where $F_{m,b}^{N}$ and $F_{m,b}^{M}$ are the fractions of aerosol number and mass for mode m at the size bin of b out of the eight size bins. $Q(x)$ is evaluated using a numerical approximation[60] with a maximum error of 1.2×10^{-7}.

The mass of a specific aerosol species at an individual size bin, $M_{s,b}$, is calculated following Eq. (3.4). The number of total aerosols at a specific size bin, N_b, is determined by Eq. (3.5).

$$M_{s,b} = \sum_{m=1}^{3} M_{s,m} \cdot F_{m,b}^{M} \quad (3.4)$$

$$N_b = \sum_{m=1}^{3} N_m \cdot F_{m,b}^{N} \quad (3.5)$$

So far, the mass and number of aerosols has been converted from the modal distribution to the sectional distribution. Now we need to calculate the aerosol averaged radius and complex refractive indices at each size bin, which are input of the Mie calculation.

7.4. Mixing rules of aerosols and their effective refractive indices

Eq. (4.1) determines the volume of each aerosol species at a specific size bin, $V_{s,b}$. The wet and core radii, $\bar{r}_{wet,b}$ and $\bar{r}_{core,b}$, of aerosols are calculated through Eq. (4.2)–(4.5).

$$V_{s,b} = M_{s,b} / \rho_s \quad (4.1)$$

$$V_{wet,b} = \sum_{s=1}^{8} V_{s,b} \quad (4.2)$$

$$V_{shell,b} = V_{wet,b} - V_{BC,b} \quad (4.3)$$

$$\bar{r}_{wet,b} = \left(\frac{3}{4\pi}\frac{V_{wet,b}}{N_b}\right)^{1/3} \quad (4.4)$$

$$\bar{r}_{core,b} = \left(\frac{3}{4\pi} \frac{V_{BC,b}}{N_b} \right)^{1/3} \tag{4.5}$$

where $V_{wet,b}$, $V_{shell,b}$, and $V_{BC,b}$ are respectively volumes of all aerosol species, all aerosols species but BC, and BC.

Mie code is used to calculate the optical properties of aerosols. There are three assumptions of mixing rules of aerosols: volume averaging, Maxwell-Garnett, and shell-core. The volume averaging method assumes the internal-mixing of aerosol compositions, which averages the refractive indices of all aerosol species weighted by their volumes at each size bin.

$$\tilde{n}_{\lambda,b} = \frac{\sum_{s=1}^{8} \tilde{n}_{s,\lambda} \cdot V_{s,b}}{\sum_{s=1}^{8} V_{s,b}} \tag{4.6}$$

where $\tilde{n}_{\lambda,b}$ is the effective complex refractive index of all aerosol species at wavelength λ and bin b, and λ is 300, 400, 600, and 999 nm for shortwave, and 3.5, 4.0, 4.3, 4.6, 5.2, 6.2, 7.0, 7.9, 8.9, 9.7, 11.1, 13.2, 15.1, 17.9, 24.3, and 514.3 μm for long-wave.

The shell-core method assumes a core composed of BC, which is surrounded by a shell composed of all other aerosol compositions.

$$\tilde{n}_{\lambda,shell,b} = \frac{\sum_{s=1}^{8} \tilde{n}_{s,\lambda} \cdot V_{s,b} - \tilde{n}_{BC,\lambda} \cdot V_{BC,b}}{V_{shell,b}} \tag{4.7}$$

$$\tilde{n}_{\lambda,core} = \tilde{n}_{\lambda,BC} \tag{4.8}$$

The Maxwell-Garnett method assumes that BC randomly distributes within an aerosol particle. The effective refractive index is determined by Eqs. (4.9) and (4.10) [61].

$$f_b = \left(\frac{\bar{r}_{core,b}}{\bar{r}_{wet,b}} \right)^3 \tag{4.9}$$

$$\tilde{n}_{\lambda,b} = \tilde{n}_{\lambda,shell,b} \cdot \left[1 + \frac{3 f_b \left(\tilde{n}_{\lambda,core}^2 - \tilde{n}_{\lambda,shell,b}^2 \right)}{\left(1 - f_b\right) \tilde{n}_{\lambda,core}^2 + \left(2 + f_b\right) \tilde{n}_{\lambda,shell,b}^2} \right]^{1/2} \tag{4.10}$$

where f_b is the volume fraction of the core to the whole aerosol particle.

7.5. Aerosol optical properties

The Mie code takes in aerosol number concentration (N_b), radius ($\bar{r}_{wet,b}$ and $\bar{r}_{core,b}$), and complex refractive indices ($\tilde{n}_{\lambda,b}$ or $\tilde{n}_{\lambda,shell,b}$ and $\tilde{n}_{\lambda,core}$) at each wavelength and bin to calculate the aerosol extinction efficiency (ε_λ), scattering efficiency (ϖ_λ), asymmetry parameter (g_λ), and backscattering efficiency (b_λ) of a single particle. Multiplying the optical efficiencies of single aerosol particle with aerosol number concentration at a specific size bin and then summing these multiplications over the eight bins gives the aerosol optical properties at a specific wavelength, as formulated by Eqs. (5.1)–(5.5).

$$\varepsilon_\lambda = \sum_{b=1}^{8} \varepsilon_{\lambda,b} \cdot N_b \tag{5.1}$$

$$\varpi_\lambda = \frac{\sum_{b=1}^{8} \omega_{\lambda,b} \cdot N_b}{\varepsilon_\lambda} \tag{5.2}$$

$$g_\lambda = \frac{\sum_{b=1}^{8} g_{\lambda,b} \cdot \omega_{\lambda,b} \cdot N_b}{\sum_{b=1}^{8} \omega_{\lambda,b} \cdot N_b} \tag{5.3}$$

$$b_\lambda = \sum_{b=1}^{8} b_{\lambda,b} \cdot N_b \tag{5.4}$$

$$\tau_\lambda = \varepsilon_\lambda \cdot \Delta z \tag{5.5}$$

where τ_λ and Δz are respectively aerosol optical depth at a specific model layer and the depth of the this model layer. Finally, τ_λ, ϖ_λ, and g_λ are passed to RRTMG shortwave and long-wave radiation scheme to determine aerosol's direct effect.

The terms in the left-hand side of Eqs (5.1)–(5.5) represent aerosol optical properties at the wavelength of λ in each model box. The column-integrated aerosol optical properties are determined following Eqs (5.6) and (5.7). For SSA, which is assumed homogenous in each model layer, the column-integrated value is calculated by weighting the extinction coefficients[62], σ_i, in the entire atmospheric column to make them comparable to satellite and AERONET observations, as shown in Eq (5.6). For AOD, the column-integrated value is the summation of all τ_λ in the entire atmospheric column, as shown in Eq (5.7).

$$SSA_\lambda = \frac{\sum_{i=1}^{i=30} \varepsilon_{\lambda,i}\varpi_{\lambda,i}}{\sum_{i=1}^{i=30} \varepsilon_{\lambda,i}} \tag{5.6}$$

$$AOD_\lambda = \sum_{i=1}^{30} \tau_{\lambda,i} \tag{5.7}$$

where i is the aerosol optical properties at the ith model layer. For additional information about the representation of aerosol's direct effect in climate model, please refer to [63,64].

References

45. Shettle, E. P. & Fenn, R. W. Models for the aerosols of the lower atmosphere and effects of humidity variation on their optical properties. *Air Force Geophysics Laboratory Tech. Rep., AFGL-TR-79-0214*, 99, (1979).
46. Yi, B. Q., Hsu, C. N., Yang, P. & Tsay, S. C. Radiative transfer simulation of dust-like aerosols: Uncertainties from particle shape and refractive index. *J Aerosol Sci* **42**, 631-644, (2011).
47. Colarco, P., da Silva, A., Chin, M. & Diehl, T. Online simulations of global aerosol distributions in the NASA GEOS-4 model and comparisons to satellite and ground-based aerosol optical depth. *J Geophys Res-Atmos* **115**, D14207, (2010).
48. Hess, M., Koepke, P. & Schult, I. Optical properties of aerosols and clouds: The software package OPAC. *B Am Meteorol Soc* **79**, 831-844, (1998).
49. Perlwitz, J., Tegen, I. & Miller, R. L. Interactive soil dust aerosol model in the GISS GCM 1. Sensitivity of the soil dust cycle to radiative properties of soil dust aerosols. *J Geophys Res-Atmos* **106**, 18167-18192, (2001).
50. Wagner, R. *et al.* Complex refractive indices of Saharan dust samples at visible and near UV wavelengths: a laboratory study. *Atmos Chem Phys* **12**, 2491-2512, (2012).
51. Zhao, C., Liu, X., Leung, L. R. & Hagos, S. Radiative impact of mineral dust on monsoon precipitation variability over West Africa. *Atmos Chem Phys* **11**, 1879-1893, (2011).
52. Zhao, C. *et al.* The spatial distribution of mineral dust and its shortwave radiative forcing over North Africa: modeling sensitivities to dust emissions and aerosol size treatments. *Atmos Chem Phys* **10**, 8821-8838, (2010).
53. Colarco, P. R. *et al.* Impact of radiatively interactive dust aerosols in the NASA GEOS-5 climate model: Sensitivity to dust particle shape and refractive index. *J Geophys Res-Atmos* **119**, 753-786, (2014).
54. Colarco, P. R., Toon, O. B., Torres, O. & Rasch, P. J. Determining the UV imaginary index of refraction of Saharan dust particles from Total Ozone Mapping Spectrometer data using a three-dimensional model of dust transport. *J Geophys Res-Atmos* **107**, AAC 4-1–AAC 4-18, (2002).
55. Kim, D. *et al.* Dust optical properties over North Africa and Arabian Peninsula derived from the AERONET dataset. *Atmos Chem Phys* **11**, 10733-10741, (2011).
56. Haapanala, P., Raisanen, P., Kahnert, M. & Nousiainen, T. Sensitivity of the shortwave radiative effect of dust on particle shape: Comparison of spheres and spheroids. *J Geophys Res-Atmos* **117**, D08201, (2012).
57. Kinne, S. *et al.* Monthly averages of aerosol properties: A global comparison among models, satellite data, and AERONET ground data. *J Geophys Res-Atmos* **108**, AAC 3-1–AAC 3-42, (2003).
58. Raisanen, P. *et al.* Impact of dust particle non-sphericity on climate simulations. *Q J Roy Meteor Soc* **139**, 2222-2232, (2013).
59. Sokolik, I. N. & Toon, O. B. Incorporation of mineralogical composition into models of the radiative properties of mineral aerosol from UV to IR wavelengths. *J Geophys Res-Atmos* **104**, 9423-9444, (1999).
60. Press, W. H., Teukolsky, S. A., Vetterling, W. T. & Flannery, B. P. Numerical Recipes in Fortran 77 The Art of Scientific Computing. Ch. 6, 214 (Cambridge University Press, 1992).

61. Levy, O. & Stroud, D. Maxwell Garnett theory for mixtures of anisotropic inclusions: Application to conducting polymers. *PHYSICAL REVIEW B* **56**, 12, (1997).
62. Curci, G. *et al.* Uncertainties of simulated aerosol optical properties induced by assumptions on aerosol physical and chemical properties: An AQMEII-2 perspective. *Atmos Environ* **115**, 541-552, (2015).
63. Fast, J. D. *et al.* Evolution of ozone, particulates, and aerosol direct radiative forcing in the vicinity of Houston using a fully coupled meteorology-chemistry-aerosol model. *J Geophys Res-Atmos* **111**, D21305, (2006).
64. Ghan, S. *et al.* Evaluation of aerosol direct radiative forcing in MIRAGE. *J Geophys Res-Atmos* **106**, 5295-5316, (2001).
